# Water Level Flux in Household Containers in Vietnam - A Key Determinant of *Aedes aegypti* Population Dynamics

**DOI:** 10.1371/journal.pone.0039067

**Published:** 2012-07-24

**Authors:** Jason A. L. Jeffery, Archie C. A. Clements, Yen Thi Nguyen, Le Hoang Nguyen, Son Hai Tran, Nghia Trung Le, Nam Sinh Vu, Peter A. Ryan, Brian H. Kay

**Affiliations:** 1 Queensland Institute of Medical Research, PO Royal Brisbane Hospital, Brisbane, Queensland, Australia; 2 Infectious Disease Epidemiology Unit, School of Population Health, University of Queensland, Herston, Queensland, Australia; 3 National Institute of Hygiene and Epidemiology, Hanoi, Vietnam; 4 Institute Pasteur, Nha Trang, Vietnam; 5 General Department of Preventive Medicine and Environmental Health, Ministry of Health, Hanoi, Vietnam; Centro de Pesquisas René Rachou, Brazil

## Abstract

We examined changes in the abundance of immature *Aedes aegypti* at the household and water storage container level during the dry-season (June-July, 2008) in Tri Nguyen village, central Vietnam. We conducted quantitative immature mosquito surveys of 171 containers in the same 41 households, with replacement of samples, every two days during a 29-day period. We developed multi-level mixed effects regression models to investigate container and household variability in pupal abundance. The percentage of houses that were positive for I/II instars, III/IV instars and pupae during any one survey ranged from 19.5–43.9%, 48.8–75.6% and 17.1–53.7%, respectively. The mean numbers of *Ae. aegypti* pupae per house ranged between 1.9–12.6 over the study period. Estimates of absolute pupal abundance were highly variable over the 29-day period despite relatively stable weather conditions. Most variability in pupal abundance occurred at the container rather than the household level. A key determinant of *Ae. aegypti* production was the frequent filling of the containers with water, which caused asynchronous hatching of *Ae. aegypti* eggs and development of cohorts of immatures. We calculated the probability of the water volume of a large container (>500L) increasing or decreasing by ≥20% to be 0.05 and 0.07 per day, respectively, and for small containers (<500L) to be 0.11 and 0.13 per day, respectively. These human water-management behaviors are important determinants of *Ae. aegypti* production during the dry season. This has implications for choosing a suitable *Wolbachia* strain for release as it appears that prolonged egg desiccation does not occur in this village.

## Introduction

Dengue affects 50 million people annually with approximately 20,000 deaths [1]. Four antigenically related but distinct viruses are transmitted principally by the mosquito *Aedes aegypti* (L.). Because there is no vaccine available, vector control remains the cornerstone of epidemic prevention and control [2], [3].

Control of *Ae. aegypti* using virulent strains of *Wolbachia* has gained impetus due to life-shortening [4] and/or viral interference [5] phenotypes observed after successful micro-injection of *Ae. aegypti* with *w*Mel and *w*MelPop strains in the laboratory. In the summer of 2011, *w*Mel-infected *Ae. aegypti* adults were successfully released into two field localities around Cairns, Australia [6], demonstrating the feasibility of *Wolbachia*-based dengue control strategies under field conditions.

To trial a *Wolbachia*-based control strategy in Vietnam, the village of Tri Nguyen has been selected by the Ministry of Health as a potential release site for *w*MelPop-CLA (a mosquito cell-line adapted isolate of *w*MelPop) transinfected *Ae. aegypti*. This village is located on Hon Mieu Island, approximately 1 km off the coast of central Vietnam. This *w*MelPop-CLA strain causes several fitness effects on *Ae. aegypti* [3] including reduced fecundity due to life-shortening and reduced ability of eggs to withstand desiccation [7], two phenotypes that could hinder the establishment of the wMelPop-CLA transinfected *Ae. aegypti*, particularly over the dry season. It has been suggested that in tropical areas such as Thailand and Vietnam, where abundant breeding sites are regularly filled by rainfall and/or by human manipulation, the *w*MelPop-CLA strain may spread and persist in *Ae. aegypti* [7]. Thus one of the goals of this paper was to examine the effect of human water manipulation behaviours on *Ae. aegypti* populations in this village during the dry season.

From the results of nine entomologic surveys conducted over 14 months, we determined that village-wide spatial patterns in *Ae. aegypti* presence and abundance in houses were considerably heterogeneous [8]. Importantly, key premises were present with high numbers of mosquitoes, although in contrast to North Queensland [9] and Trinidad [10], these were not temporally stable. In Vietnam, the pattern observed over 14 months suggested that at the household level, *Ae. aegypti* production displayed a cohort or pulse effect, rather than production being continuous and overlapping between mosquito generations. Surprisingly, there was no clear association between season and the prevalence or abundance of *Ae. aegypti* immatures (larvae or pupae) or adults, even though central Vietnam experiences distinct wet (September – December) and dry seasons (February – August) [8]. This lack of a clear association between season and *Ae. aegypti* abundance was noted in the analysis of long-term *Ae. aegypti* data from Puerto Rico and Thailand. Mosquito populations in those two countries were sensitive to different environmental factors (rainfall in Puerto Rico, temperature in Thailand), probably a reflection of local habitat differences and adaptation to unique seasonal environments (distinct wet- and dry-seasons in Thailand, but not in Puerto Rico) [11].

Our study aimed to examine the abundance of *Ae. aegypti* in a village in central Vietnam during the dry season in relation to householder water storage management. Specifically, we wanted to know how frequently householders were filling or emptying their containers, and whether this was sufficient to maintain *Ae. aegypti* populations during a period when little rainfall was expected. This information will be useful in a control program based on the use of *w*MelPop-CLA transinfected *Ae. aegypti*, which has a phenotypic disadvantage whereby the eggs have reduced desiccation resistance [7]. We sampled a cohort of 171 containers at 41 houses, measuring *Ae. aegypti* production every 2 d for almost a month and recording water volume changes in containers. We then used multi-level models to determine whether household- or container-level factors contributed more to *Ae. aegypti* abundance.

## Materials and Methods

### Ethics

All necessary permits were obtained for the described field studies. Informed verbal consent was obtained from the head of each household according to the Institute Pasteur Nha Trang ethics policy. Although samples had to be returned to the containers after each survey, all mosquito immatures were discarded after the survey on day 29.

### Study Site

The area chosen for this study was the village of Tri Nguyen, on Hon Mieu island (12^o^18’N, 109^o^14’E), Khanh Hoa province, central Vietnam. A description of the features of the village, cultural practices, occupations and a map, can be found in a report of our long-term entomological survey of this island [8]. In 9 surveys from November 2006–December 2007, large water storage containers (moulded tanks, cylindrical tanks, box tanks and large jars) contained 97–100% of the standing crop of third and fourth instars, and 93–100% of pupae. Small containers such as discards, vases and ant traps contained <5% of production. House type, education level, occupation, income and water management behavior were recorded by the survey staff at this time.

### Entomologic Surveys

The current study was undertaken in June-July 2008 to define the short-term temporal variability in immature *Ae. aegypti* production at the household and container level. Forty-one houses (6.7%) were randomly selected from a geo-referenced database containing information on the 611 houses in Tri Nguyen village [8]. These houses were then surveyed every 2 d, for a total of 29 d, by two teams of 2–3 people. The 2-day sampling period was chosen as it matched the minimum duration of the pupal stage [8], [12] so that cohorts were not missed. On the day of the first survey, containers located in and around each house were marked with a unique identification number so these could be tracked throughout the study, and any new ones recognized. Every 2 d, the volume, source and use of water, location of the container, and lid status (full, partial or no cover) was recorded for each container. All wet containers were then sampled (either with the 5 sweep net method or pipette) [13]. For both *Aedes* and *Culex* spp. immatures, presence/absence of I/II instars and the approximate number of III/IV instars (0, 1–10, 11–100, 101–1000, 1000+) were recorded. The number of pupae were counted. Presence or absence of potential mosquito predators (principally *Mesocyclops* spp., *Micronecta* spp. and fish) was also recorded. The sample was then returned to the container. On each sampling occasion, the height of the water level in each container was estimated using a graduated rule. The height was then used to calculate the volume of water in each container and this was expressed as a percentage of the total capacity. Percentage change between successive surveys was used to measure water flux.

Because of the large size and configuration of the containers, plus their usage patterns [13], it was not possible to estimate oviposition and egg hatching using paper strips [14], [15]. Consequently, a modelling approach was adopted using pupal counts. We assumed that the main factor influencing pupal abundance is water level changes triggering egg hatching, although we acknowledge that unmeasured variables such as overcrowding, nutrient levels and egg-laying behaviours can influence immature abundance. We also acknowledge that water-level changes may have occurred without our knowledge in the period between visits every 2 d and that these may have also influenced immature abundance.

**Figure 1:**
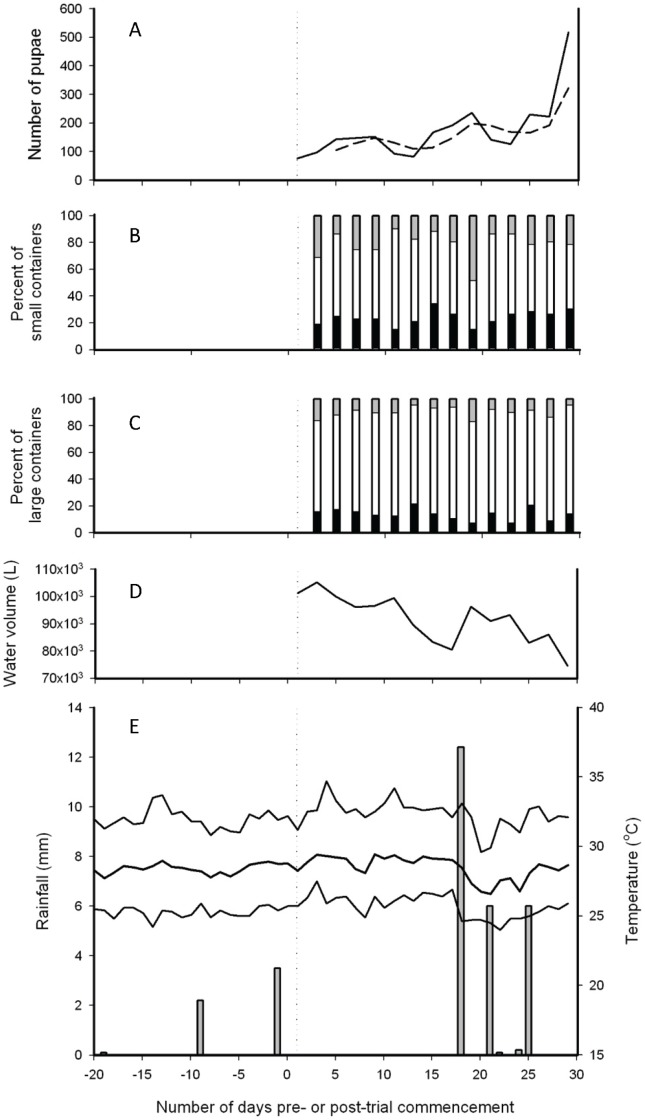
Absolute counts of pupae every 2 days, in relation to water flux, water storage and weather conditions.

For the water volume analyses, 171 containers within the 41 properties were classified as either large (>500 L, n = 119) or small (<500 L, n = 52). A variable was created to indicate water flux, categorized according to whether the volume of water had increased by >20%, decreased by >20% or neither increased or decreased by >20%, relative to the previous survey. Daily rainfall (mm) and daily minimum, mean and maximum temperatures (°C) were obtained from Nha Trang city weather station (Figure 1). June/July is the dry and hot season in central Vietnam and there were only three rain events >5 mm during the survey period.

### Multi-level Model of Pupal Abundance

Pupal abundance was highly aggregated because a large proportion (range 76.8–94.8%) of the containers during any one survey was negative for pupae. We investigated different types of models of the pupal counts, including a Poisson model, a negative binomial model and a zero-inflated Poisson (ZIP) model and found that the ZIP model provided the best fit to the data (using the Akaike Information Criterion and the Vuong statistic). Consequently, we used a ZIP mixed effects model to examine the relationship between water volume changes and pupal abundance. This allowed us to incorporate both the Poisson structure of the distribution of pupal counts (which includes some zeroes) and a zero-inflated component that modelled the excess negative containers.

The model took the form:
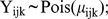


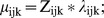


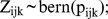


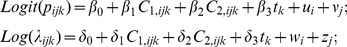
where *Y* was the observed number of pupae in container *i,* household *j,* survey *k*, *μ* was the modelled mean number of pupae, *Z* represented the excess zeroes in the observed pupal distribution, and *λ* the count of pupae. For the zero-inflated part of the model, *β_0…2_* were the intercept and coefficients for the fixed effects (two dummy variables representing categories of water level change: C_1_ an increase in water volume ≥20% relative to the last survey and C_2_, a decrease in water volume ≥20% relative to the last survey, with the reference category being a change in water level of <20%, and a term for temporal trend), *u_i_* was a container-level random effect and *v_j_* was a household-level random effect. Similarly, for the count part of the model, *δ_0…2_* were the intercept and coefficients for the fixed effects (as above), *w_i_* was a container-level random effect and *z_j_* was a household-level random effect. Because change in container water volume was assessed relative to the previous survey, we only used data from surveys 2–15 in the models. We specified non-informative priors for the intercepts and coefficients (normal priors with a mean of 0 and a precision, the inverse of variance, of 1/10,000). All of the random effects were assumed to have a normal distribution centred on zero, and with an unknown precision modelled with non-informative gamma priors (having shape and scale parameters  = 0.01).

We used a Markov chain Monte Carlo simulation to fit the models. A burn-in of 5,000 iterations was allowed, followed by 100,000 iterations where values for the intercept, coefficients, and the means and variance of the random effects were monitored and stored. To reduce autocorrelation in the chains, only every 10^th^ iteration was stored, giving a total of 10,000 iterations for the posterior distribution of each monitored variable. Convergence was checked by visual examination of density and history plots. To ensure that sufficient iterations were performed to adequately describe the posterior distributions, Monte Carlo error (MCE)/SD was calculated for each variable. If this value was <0.05 for each parameter, we considered the number of iterations to be sufficient [16]. Random effects were considered significant at the 5% level when the 95% credible intervals excluded zero. All analyses were performed using WinBUGS version 1.4 (Imperial College, London, and Medical Research Council, UK).

## Results

### Descriptive Analyses

The mean number of people per house was 5.6 (range 1–13). Water was stored in containers ranging in size from <100–10,000 L. Containers were replenished by purchasing water or by channelling rain water from the roof into selected containers. There were 195 containers identified in the 41 households (mean of 4.8 per household) of which 171 were examined at every time-point (i.e. 15 times). The remaining 24 containers were excluded from further analyses because they were not surveyed 15 times. This was due to householders switching their containers to non-water storage uses at some stage during the survey period. Any residual water was usually poured into another container. There were no new containers introduced during the study period.

Of the 171 containers, 17 (9.9%) were moulded tanks (2000 L capacity), 91 (53.2%) were cylindrical tanks (1000–2000 L capacity), 11 (6.4%) were box tanks (100–10,000 L capacity), 42 (24.6%) were standard jars and drums (>100 L capacity), and 7 (5.0%) were small jars (including buckets) (<100 L capacity). Almost all of the containers (98%) were located outdoors with those indoors mainly used during routine daily activity. As with previous surveys [8], numbers of predators were low, ranging from 0–4%.

### Temporal Patterns

The total estimated number of pupae collected from all houses ranged from 77 on day 1 (mean of 1.9 per house) to 517 on day 29 (mean of 12.6 per house) (Figure 1). The percentage of containers that were wet during each survey ranged from 72.8–82.1%. The trend in volume of water stored over the 29 days was generally decreasing (a reduction from 102,000 to 75,000 L) but regular water replenishment was evident in both large and small containers (Figure 1). Increases of >20% in the volume of water in containers, compared with water volume observed during the previous survey period, could be seen during all sampling periods. This was due to householders either purchasing water from vendors or by consolidating stored water into fewer containers. Two of the three rain events on days 21 (5.6 mm) and 25 (6.1 mm) had a negligible effect on the filling of containers (only 13% of small and 8% of large containers had increases in water volumes of ≥20% on day 21, and 21% of small and 8% of large containers increased by ≥20% on day 25). However, the rainfall event on day 18 (12.5 mm) resulted in the highest rates of container filling, with increases in water volumes ≥20% in 48% of small containers and 17% of large containers. The latter event resulted in a 2.3 to 6.6-fold rise in pupal abundance 11 d later, from between 1.9 and 5.6 pupae per house on days 1–18, to 12.6 pupae per house on day 29.

In terms of container-level water volume changes observed during each survey, there were 304 events where water volume increased ≥20%, 410 events where water volume decreased ≥20%, and 1851 events in which water volumes did not vary by ≥20%, compared to the previous survey. Water volume changes occurred every day, with a range of 9.6–48.1% of small containers being filled and 15.4–34.6% being reduced by ≥20%, respectively, and 4.2–16.8% and 7.6–21.8% of large containers being filled or reduced by ≥20%, respectively. The percentage of small and large containers whose volume did not change by ≥20% compared with the previous survey, ranged from 36.5–75.0% and 68.0–83.2%, respectively (Figure 1). The probability of the water volume of a large container (>500 L) increasing ≥20% was 0.05 and decreasing ≥20% was 0.07 per day. For small containers (<500 L) it was 0.11 (increasing) and 0.13 (decreasing) per day.

The percentage of houses and containers that was positive for pupae during any one survey ranged from 17.1–53.7 and 5.2–21.4%, respectively. However, by the end of the 29 d, 87.8% of houses and 46.2% of containers had been recorded as positive (Table 1), indicating that even in a short time frame, most houses and almost half of all containers had or were producing pupae. Similar patterns were observed for I/II and III/IV instars. Interestingly, seven (17.1%) houses were positive for III/IV instars at every time point but no houses were consistently positive for I/II instars or pupae. Only one container was consistently positive for III/IV instars at every time point and no containers were consistently positive for I/II instars or pupae. There were also a proportion of houses (5%) and containers (40%) that remained consistently negative for immature *Ae. aegypti* throughout the entire study (Table 1).

**Table 1:** Percentage of houses and containers positive or negative for immature *Ae. Aegypti.*

	Houses (%)			Containers (%)		
	Range +ve during the surveys	Cumulative +ve	Always –ve	Range +ve duringthe surveys	Cumulative +ve	Always –ve
I/II instars	19.5–43.9	90.2	9.8	6.3–20.0	56.1	43.9
III/IV instars	48.8–75.6	92.7	7.3	20.3–37.0	63.7	36.3
Pupae	17.1–53.7	87.8	12.2	5.2–21.4	46.2	53.8
All stages			5.0			40.0

### Statistical Analysis

The Bayesian hierarchical model showed that there was more variation in pupal abundance at the container level compared with the house level for both the zero-inflated and count components of the model (Table 2). Containers where the water volume increased relative to the previous survey had a significantly higher count of pupae (if pupae were present) and the counts of pupae showed a significantly increasing trend over the study period.

**Table 2:** Results from the analysis of *Ae. aegypti* pupal abundance using a zero-inflated Poisson model in a Bayesian framework.

Variable	Zero-inflated component	Count component
Intercept	–3.58 (–4.18– –3.02)	1.45 (1.17–1.72)
Coefficient: increasing volume	–0.48 (–1.04–0.06)	0.95 (0.79–1.11)*
Coefficient: decreasing volume	0.16 (–0.30–0.60)	0.06 (–0.07–0.19)
Coefficient: temporal trend	0.04 (–9.9×10^−4^–0.08)	0.06 (0.04–0.07)*
Variance container RE	2.43 (1.50–4.14)	0.78 (0.53–1.24)
Variance household RE	0.06 (0.01–1.19)	0.03 (0.01–0.32)

* Significant with ≥95% probability; RE = random effect; estimates show the mean and 95% Bayesian credible interval.

A significant container-level random effect indicated that there were unmeasured variables acting at the container level that influenced pupal presence and abundance (note, values of the random effects are not shown). For the zero-inflated component of the model, no containers had random effects significantly lower than the overall mean and 21 containers had random effects significantly greater than the overall mean (i.e. they were more likely than average to have a zero count). For the count component of the model, 5 containers had random effects significantly lower than the overall mean and 20 containers had random effects significantly greater than the overall mean (i.e. they had a significantly higher than average count). None of the household-level random effects were significantly different from the mean (they all had 95% Bayesian credible interval limits that included zero).

## Discussion

Because piped water was unavailable, villagers in Tri Nguyen relied on water management, water purchase and occasionally rainfall to fill a variety of containers ranging from 100–10,000 L. Not surprisingly, our data provide evidence that frequent filling of containers is positively associated with the abundance of *Ae. aegypti* pupae. Despite the relative lack of rainfall and a reducing but fluctuating water volume, there were enough filling events (304 over 29 days) to support hatching of the desiccation-resistant eggs of *Ae. aegypti* inside these containers and thus ensure survival and, sometimes, population growth, although the most significant increase in pupal abundance followed 12.5 mm of rainfall on day 18. Overall, this suggests that if *w*MelPop-CLA transinfected *Ae. aegypti* are released in Tri Nguyen village, there will be sufficient water filling events from everyday householder behaviours to minimise the effect of the reduced desiccation resistant phenotype. Thus, this might support the release of *w*MelPop-CLA mosquitoes during the wet season, and their survival through the dry season.

Over the 14-month study period reported in our previous work, the percentage of houses positive for III/IV instars and pupae ranged from 54–81 and 13–48%, respectively [8]. This is similar to the equivalent ranges we observed over the one-month period of the current study (49–76 and 17–54%, respectively). In terms of container positivity for III/IV instars and pupae, the 14-month range (26–49 and 6–22%, respectively) was similar to the one-month range found in the current study (20–37 and 5–21%, respectively).

Stoddard [17] indicated that human behavior is an under-studied aspect of vector control and disease management. Our work suggests that water storage behavior, particularly in relation to human-driven water volume changes at the container-level and over small temporal scales, is an important driver of *Ae. aegypti* population dynamics. Although other studies have been undertaken on water storage practices and behaviors in Vietnam, these have focussed primarily on changes in human perceptions of water supply and changes in water storage behavior subsequent to the provision of new water supply infrastructure [18]. Our study was concerned with existing infrastructure and cultural practices of water storage. Upon questioning of householders after changes in water level ≥20%, it was apparent that we had observed a continuous practice of householder transfer of water, and not surprisingly, asynchronous hatching of *Ae. aegypti* eggs and subsequent development of cohorts of immatures. The relatively low percentage of immature positivity on any one day for houses and, to a lesser extent, containers, compared to the high cumulative house or container positivity at the end of the 29 day period, is indicative of the asynchrony of *Ae. aegypti* cohorts across the village.

The frequency of container filling events (n = 304) was 26% fewer than water draw-down events (n = 410). Although we selected 20% as a definite and observable water volume change, we acknowledge that water level increases smaller than this would also be capable of causing egg hatching, indicating one limitation of the study. We chose 20% as a cut-off because we were evaluating broad patterns of water management, and whether or not these were sufficient to maintain *Ae. aegypti* populations during a period when little rainfall was expected. Since we observed that there were enough water volume changes to support *Ae. aegypti* populations, any smaller changes that we may have overlooked would most likely have an additive effect and so our measurements of water flux are most likely underestimates. Hatching could even be initiated by disruption of the water surface during the retrieval process by householders, but this also could not be measured with any precision. Despite this, it appears unlikely that *Ae. aegypti* relies on prolonged desiccation resistance at Tri Nguyen through the dry season.

Although further investigation of this effect is required, it would seem that such behavior should be incorporated into *Ae. aegypti* population models such as CIMSiM [19], [20] and Skeeter Buster [21] to ensure they are realistic, accurate and location specific. This concurs with findings in Colombia [22] and Puerto Rico [23]. Given that more variability in pupal abundance occurred at the container level, any pre-release vector control needs to focus on all containers in the target area, and not just on key containers or high-mosquito burden households, because we saw little evidence for their existence. As in Iquitos [24], high productivity, whether in containers or households, was transient. Our data also suggest that in Tri Nguyen village, container level variability was more important than household level data. As in Iquitos [24], we have previously demonstrated that the correlation between pupal and adult abundance is the strongest [25], so we believe our estimates are robust. Although we acknowledge that we only studied a small number of houses, our models also suggest that there were significantly more unmeasured variables at the container level influencing pupal abundance, compared to the household level. We expect that this would be applicable to other immature stages. Thus as with other studies [22], [23], human water management practices would seem to be a previously underrated factor in driving container productivity of *Ae. aegypti*.

## References

[1] Beatty ME, Beutels P, Meltzer MI, Shepard DS, Hombach J (2011). Health economics of dengue: a systematic literature review and expert panel’s assessment.. Am J Trop Med Hyg.

[2] Gubler D, Gubler D, Kuno G (1997). Dengue and dengue hemorrhagic fever: its history and resurgence as a global public health problem..

[3] Iturbe-Ormaetxe I, Walker T, O’Neill SL (2011). *Wolbachia* and the biological control of mosquito-borne disease.. EMBO Rep.

[4] McMeniman CJ, Lane RV, Cass BN, Fong AW, Sidhu M (2009). Stable introduction of a life-shortening *Wolbachia* infection into the mosquito *Aedes aegypti*.. Science.

[5] Moreira LA, Iturbe-Ormaetxe I, Jeffery JA, Lu G, Pyke AT (2009). A *Wolbachia* symbiont in *Aedes aegypti* limits infection with dengue, Chikungunya, and *Plasmodium*.. Cell.

[6] Hoffmann AA, Montgomery BL, Popovici J, Iturbe-Ormaetxe I, Johnson PH (2011). Successful establishment of *Wolbachia* in *Aedes* populations to suppress dengue transmission.. Nature.

[7] McMeniman CJ, O’Neill SL (2010). A virulent *Wolbachia* infection decreases the viability of the dengue vector *Aedes aegypti* during periods of embryonic quiescence.. PLoS Negl Trop Dis.

[8] Jeffery JAL, Yen NT, Nam VS, Nghia LT, Hoffmann AA (2009). Characterizing the *Aedes aegypti* population in a Vietnamese village in preparation for a *Wolbachia*-based mosquito control strategy to eliminate dengue.. PLoS Negl Trop Dis.

[9] Tun-Lin W, Kay BH, Barnes A (1995). Understanding productivity, a key to *Aedes aegypti* surveillance.. Am J Trop Med Hyg.

[10] Chadee DD (2004). Key premises, a guide to *Aedes aegypti* (Diptera: Culicidae) surveillance and control.. Bull Entomol Res.

[11] Chaves LF, Morrison AC, Kitron UD, Scott TW (2012). Nonlinear impacts of climatic variability on the density-dependent regulation of an insect vector of disease.. Glob Change Biol.

[12] Southwood TR, Murdie G, Yasuno M, Tonn RJ, Reader PM (1972). Studies on the life budget of *Aedes aegypti* in Wat Samphaya, Bangkok, Thailand.. Bull World Health Organ.

[13] Knox TB, Yen NT, Nam VS, Gatton ML, Kay BH (2007). Critical evaluation of quantitative sampling methods for *Aedes aegypti* (Diptera: Culicidae) immatures in water storage containers in Vietnam.. J Med Entomol.

[14] Wong J, Astete H, Morrison AC, Scott TW (2011). Sampling considerations for designing *Aedes aegypti* (Diptera:Culicidae) oviposition studies in Iquitos, Peru: substrate preference, diurnal periodicity, and gonotrophic cycle length.. J Med Entomol.

[15] Wong J, Stoddard ST, Astete H, Morrison AC, Scott TW (2011). Oviposition site selection by the dengue vector *Aedes aegypti* and its implications for dengue control.. PLoS Negl Trop Dis.

[16] Clements AC, Moyeed R, Brooker S (2006). Bayesian geostatistical prediction of the intensity of infection with *Schistosoma mansoni* in East Africa.. Parasitology.

[17] Stoddard ST, Morrison AC, Vazquez-Prokopec GM, Paz Soldan V, Kochel TJ (2009). The role of human movement in the transmission of vector-borne pathogens.. PLoS Negl Trop Dis.

[18] Tran HP, Adams J, Jeffery JAL, Nguyen YT, Vu NS (2010). Householder perspectives and preferences on water storage and use, with reference to dengue, in the Mekong Delta, southern Vietnam.. Int Health.

[19] Focks DA, Daniels E, Haile DG, Keesling JE (1995). A simulation model of the epidemiology of urban dengue fever: literature analysis, model development, preliminary validation, and samples of simulation results.. Am J Trop Med Hyg.

[20] Focks DA, Haile DG, Daniels E, Mount GA (1993). Dynamic life table model for *Aedes aegypt*i (Diptera: Culicidae): analysis of the literature and model development.. J Med Entomol.

[21] Magori K, Legros M, Puente ME, Focks DA, Scott TW (2009). Skeeter Buster: a stochastic, spatially explicit modeling tool for studying *Aedes aegypti* population replacement and population suppression strategies.. PLoS Negl Trop Dis.

[22] Padmanabha H, Soto E, Mosquera M, Lord CC, Lounibos LP (2010). Ecological links between water storage behaviors and *Aedes aegypti* production: implications for dengue vector control in variable climates.. Ecohealth.

[23] Barrera R, Amador M, MacKay AJ (2011). Population dynamics of *Aedes aegypti* and dengue as influenced by weather and human behavior in San Juan, Puerto Rico.. PLoS Negl Trop Dis.

[24] Getis A, Morrison AC, Gray K, Scott TW (2003). Characteristics of the spatial pattern of the dengue vector, *Aedes aegypti*, in Iquitos, Peru.. Am J Trop Med Hyg.

[25] Knox TB, Nguyen YT, Vu NS, Kay BH, Ryan PA (2010). Quantitative relationships between immature and emergent adult *Aedes aegypti* (Diptera: Culicidae) populations in water storage container habitats.. J Med Entomol.

